# Improvement of cognitive and motor performance with mitotherapy in aged mice

**DOI:** 10.7150/ijbs.40886

**Published:** 2020-01-16

**Authors:** Zizhen Zhao, Zhenyao Yu, Yixue Hou, Le Zhang, Ailing Fu

**Affiliations:** 1 School of Pharmaceutical Sciences, Southwest University, Chongqing 400715, China.; 2 College of Computer Science, Sichuan University, Chengdu, 610065, China.

**Keywords:** mitochondria, bioenergy, learning and memory, motor

## Abstract

Changes in mitochondrial structure and function are mostly responsible for aging and age-related features. Whether healthy mitochondria could prevent aging is, however, unclear. Here we intravenously injected the mitochondria isolated from young mice into aged mice and investigated the mitotherapy on biochemistry metabolism and animal behaviors. The results showed that heterozygous mitochondrial DNA (mtDNA) of both aged and young mouse coexisted in tissues of aged mice after mitochondrial administration, and meanwhile, ATP content in tissues increased while reactive oxygen species (ROS) level reduced. Besides, the mitotherapy significantly improved cognitive and motor performance of aged mice. Our study, at the first report in aged animals, not only provides a useful approach to study mitochondrial function associated with aging, but also a new insight into anti-aging through mitotherapy.

## Introduction

Mitochondrial dysfunction, including decreased oxidative phosphorylation capability and increased reactive oxygen species (ROS) production, is substantially responsible for aging and age-related features 1. Studies in various organisms, such as nematodes, Drosophila, rodents, and humans, have strongly supported that aging is closely associated with mitochondrial dysfunction 2,3. Thus, protection of the mitochondrial structure or stimulation of mitochondrial function is considered as practical ways in anti-aging 4,5. However, since most of the mitochondrial damage is irreversible during aging process, the agents can always provide limited protection.

Mitochondrial therapy (mitotherapy) is to transfer functional exogenous mitochondria into mitochondria-defective cells for recovery of the cell viability and consequently, prevention of the disease progress. Accumulating evidence has indicated that exogenous mitochondria can directly enter animal tissue cells for disease therapy following local and intravenous administration 6,7. In our recent reports, systemic injection of isolated mitochondria could reduce liver injury induced by acetaminophen and high-fat diet through improving hepatocyte energy supply and decreasing oxidative stress 8,9. Therefore, we assumed that the mitochondria isolated from young animals (young mitochondria) into aged ones might play a role in anti-aging.

In this study, we intravenously administrated the young mitochondria into aged mice to evaluate whether energy production increase in aged tissues or age-related behaviors improved after the mitochondrial transplantation. The study, for the first time, offer new insight for mitotherapy on aged animals and give important evidence of understanding mitochondrial function in aging.

## Results

### Supplement of young mitochondria in aged mouse tissues after mitochondrial administration

**Figure 1:**
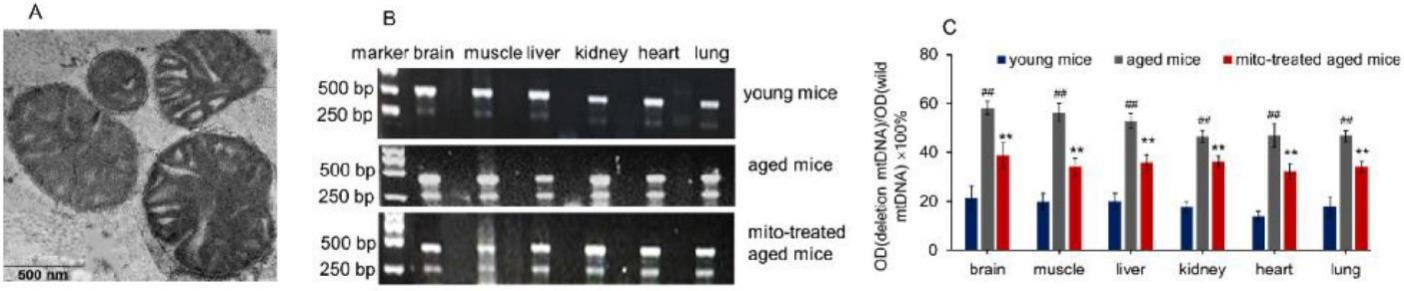
Supplementation mitochondria into tissues of aged mice. (A), the injected mitochondria were observed under TEM. (B), competitive PCR to detect the deleted mtDNA and mutant mtDNA. (C), quantitative PCR to quantify the ratio of deleted mtDNA to mutant mtDNA in brain, skeletal muscle, liver, kidney, heart, lung of aged mice. All data were expressed as the mean ± SEM. Mito, mitochondria. ^##^ *p* < 0.01 compared with the ratio of the young mice, and ^**^p < 0.01 compared with that of the aged mice. Student's *t* test was employed to compare the difference between the groups. Three independent replicates were used for each tissue.

The mitochondria were isolated from young mouse liver. Under the microscope, the mitochondria displayed spherical shape with good dispersion. Besides, double membrane structure and cristae of the isolated mitochondria remained intact, observing by TEM (Fig. 1A). It's well known that increasing age in mammals correlates with increased levels of mtDNA mutations and a deteriorating respiratory chain function, and aging-associated point mutations and deletions of mtDNA accumulate in a variety of tissues of aged animals, such as muscle, liver, lung, kidney, brain, heart, and spleen 10,11. Among mtDNA mutations of aging, 4236 bp deletion of mtDNA from nt8884 to nt13120 bp is suggested as one of the most common deletions, and the deletion proportion to wild-type mtDNA is intensely correlative with aging 12.

Here, the supplement of young mitochondria in aged mouse tissues was measured by deleted mtDNA ratio. Tissue mtDNA was respectively extracted after mitochondrial administration for competitive PCR reaction by using primers F1, F2, and R simultaneously. Under UV, almost all PCR products showed two bands that appeared at 469 bp and 256 bp (Fig. 1B), in which the 469 bp band represents as wild-type mtDNA, while the 256 bp band as deleted mtDNA. The images showed that 256 bp band was weak in tissues of young mice, while high photodensity exhibited in the aged mice (Fig. 1B). The relative content of deleted mtDNA significantly increased in aged mouse tissues compared with that of young mice (Fig. 1C). However, the photodensity of 256 bp band reduced (Fig. 1B), and the ratio of deletion and wild- type mtDNA decreased in aged tissues after the mice repeatedly received mitochondrial administration (Fig. 1C).

### Intracellular mitochondrial heteroplasmy after mitochondrial administration

**Figure 2:**
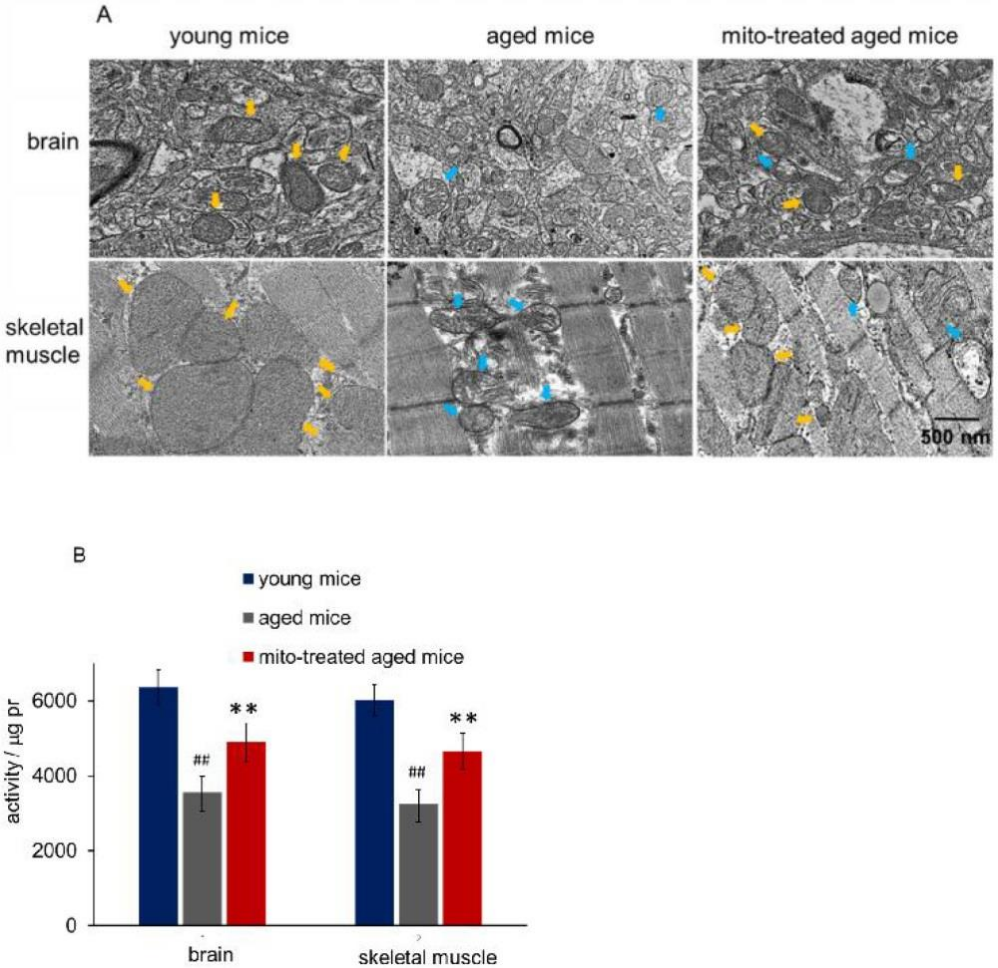
Mitochondria in brain and skeletal muscle. (A), the representative images of tissue mitochondria under TEM. The intracellular healthy mitochondrial numbers increased after mitochondrial transplantation. Yellow arrows point to healthy mitochondria, while blue arrows to aged mitochondria. (B), activities of isolated mitochondria in brain or skeletal muscle. Mito, mitochondria. The difference was analyzed by Student's *t* test. ^##^* p* < 0.01 compared to the young mice, and **p < 0.01 with the aged mice.

Tissues with high metabolism are particularly vulnerable to mitochondrial dysfunction. Encephalopathy and myopathy are common phenotypes in mitochondrial disorders. Here we used TEM to observe mitochondrial morphology of the brain and skeletal muscle, meanwhile, the mitochondria activity was measured. Brain mitochondria in young mice showed intact and parallel cristae, while mitochondria in aged mice exhibited vacuoles cavitation, shrinkage, and reduction of mitochondrial cristae (Fig. 2A). Along with the changes of mitochondrial structure, the mitochondrial activity decreased significantly (Fig. 2B). However, mitochondrial activity increased in mitochondria-treated aged mice, and the mitochondria exhibited obvious heteroplasmy with the coexistence of intact and abnormal mitochondria (Fig. 2B). In addition, mitochondrial structure damaged and activity reduced in skeletal muscle of aged mice, while intact mitochondria appeared and mitochondrial activity increased after the mice received mitochondrial administration (Fig. 2A and 2B).

### Effect of the mitotherapy on energy and redox production in the brain

**Figure 3:**
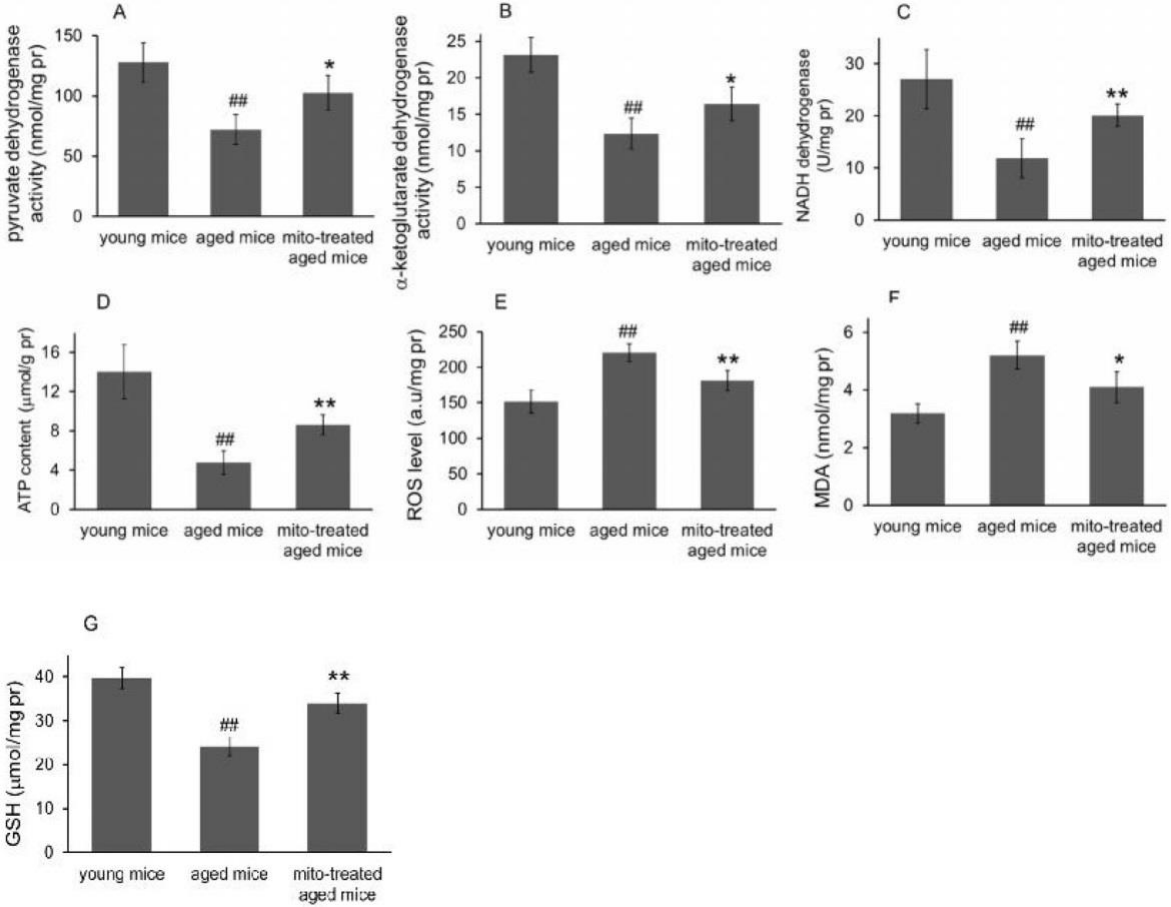
Effects of mitochondrial transplantation on bioenergy and bioredox of mouse brians. The injected mitochondria were isolated from the young mice. Activities of pyruvate dehydrogenase (A), α-ketoglutarate dehydrogenase (B), and NADH dehydrogenase (C) were respectively measured. (D), ATP content. (E), ROS level. (F) MDA content. (G), GSH content. The data were expressed as mean ± S.E.M (n = 4 for each group). The difference was analyzed by Student's *t* test. ^##^* p* < 0.01 compared to young control, and *p < 0.05, **p < 0.01 with aged group.

Because the brain is sensitive to age-related mitochondrial impairments, here we examined the mitochondria-associated biochemical properties of the brain after mitochondrial administration. Activities of mitochondrial key enzymes of aerobic oxidation, including pyruvate dehydrogenase, α-ketoglutarate dehydrogenase, and NADH dehydrogenase, decreased in aged animal brains (Fig. 3A, 3B and 3C), and these decreases were consistent with the loss of mitochondrial bioenergy production (Fig. 3D). However, mitotherapy partly recovered the enzyme activities and supplemented mitochondrial function in the energy supply of aged mice (Fig. 3A-D).

Mitochondria are not only the primary producers of energy in cells but also the main source of ROS. It has been known for a long time that dysfunctional mitochondria in aged animals are more prone to produce excessive ROS and leak it into the cytosol 13, as the result of an accumulation of damage to biomolecules causing cell injury and subsequent death. In the study, levels of ROS and MDA (peroxidation product of lipid) increased in aged mouse brain (Fig. 3E and 3F), along with a decrease of anti-oxidant GSH (Fig. 3G). However, the mitotherapy reduced ROS and MDA levels, probably because young mitochondria contain various antioxidants and can diminish excessive ROS.

### Mitotherapy improved learning and memory ability of aged mice

**Figure 4:**
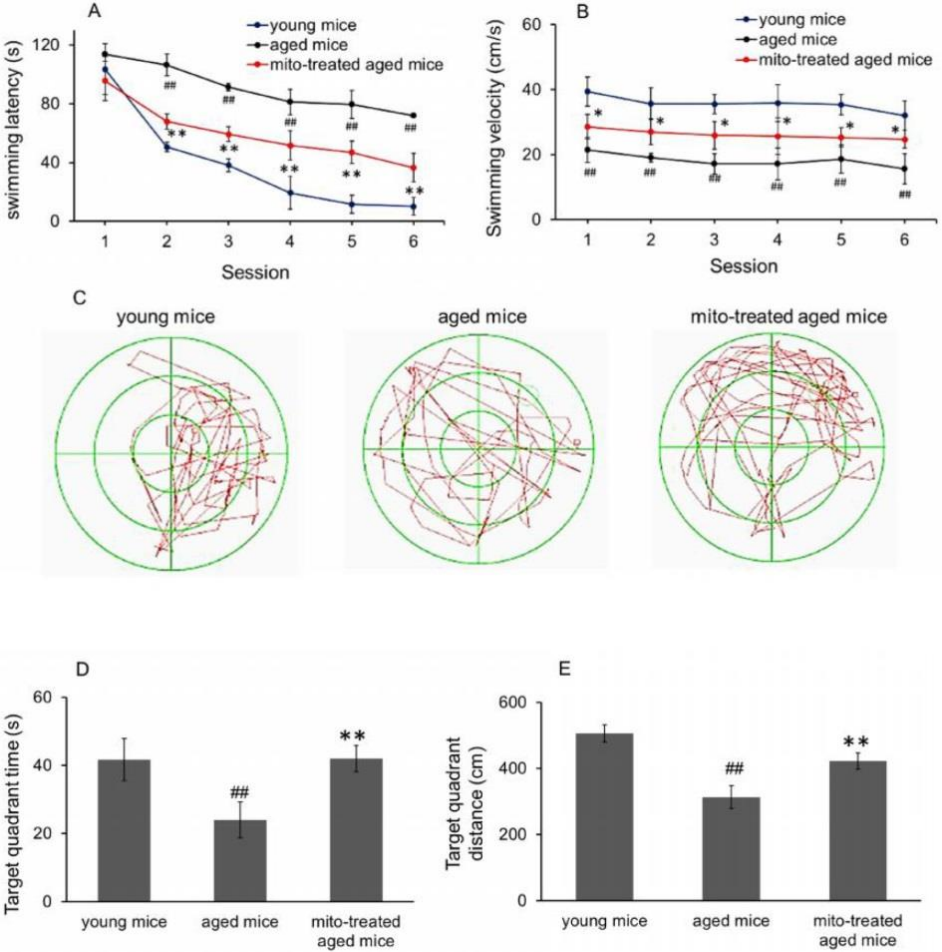
Effect of mitochondria transplantation on biochemical properties in spatial learning and memory. (A), swimming latency in navigation test. (B), swimming speed in the three groups. (C), representative swimming route of animals in probe test. In addition, time spent (D) and distance swum (E) in target quadrant increased in probe test after mitochondrial transplantation. The data were expressed as mean ± SEM (n = 10 for each group). The group difference was counted by one-way analysis of variance (ANOVA). ^##^* p* < 0.01 compared to young control, and *p < 0.05, **p < 0.01 with aged group.

To evaluate whether mitotherapy improve learning and memory ability of brain, the water maze task was used. In the navigation test, swimming latency of aged mice was longer than that of young mice from the second session, while the latency significantly decreased after mitochondrial administration (Fig. 4A). Nevertheless, there was significant difference between young mice and mitochondria-treated aged mice from the second session, suggesting that young mitochondria could improve the learning and memory ability of aged mice but cannot completely reverse the cognitive ability to the level of young mice. Meanwhile, swimming speed in the water maze task was determined. The results showed that aged mice had the slowest velocity in the three groups, and mitotherapy increased the swimming speed of aged mice (Fig. 4B), although the mice did not swim as fast as the young control. In probe test, both time spent and distance swam in target quadrant of mitochondria-treated aged mice were remarkably longer than that of aged mice (Fig. 4C, 4D and 4E), indicating that mitotherapy improved learning and memory function of aged mice.

### Enhanced skeletal muscle function after mitotherapy

**Figure 5:**
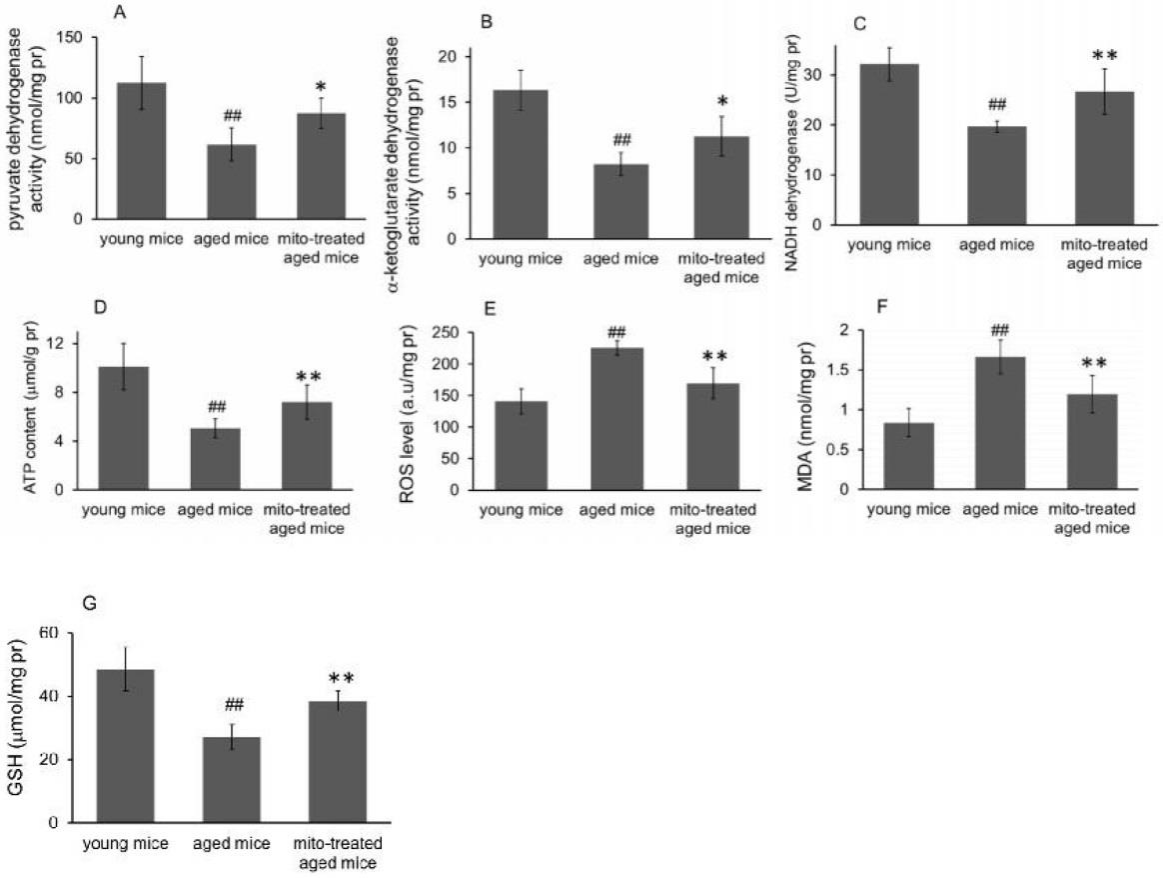
Effect of mitochondria transplantation on biochemical properties in mouse skeletal muscle. (A), pyruvate dehydrogenase; (B), α-ketoglutarate dehydrogenase; (C), NADH dehydrogenase; (D), ATP content; (E), ROS level; (F), MDA content; (F), GSH content. The data were expressed as mean ± SEM (n = 4 for each group). The difference was analyzed by Student's *t* test. ^##^* p* < 0.01 compared to young control, and *p < 0.05, **p < 0.01 with aged group.

Hypofunction of skeletal muscle is one of the most presenting features of aging. Loss of muscle strength with age is closely associated with energy deficiency 14. Under TEM, broken mitochondria showed in the skeletal muscles of aged mice, and residual mitochondria exhibited vacuoles and cristae abnormalitie (Fig. 2). Enzyme activities of mitochondrial pyruvate dehydrogenase, α-ketoglutarate dehydrogenase, and NADH dehydrogenase, significantly decreased in skeletal muscles of aged mice (Fig. 5A, 5B and 5C), and correspondingly, ATP level decreased (Fig. 5D). Moreover, ROS and MDA levels in skeletal muscles of aged mice increased while GSH content reduced. However, administration of young mitochondria reversed the changes of energy deficiency and redox condition, implying that the sport organ would have higher energy supply than aged control in physical endurance tests.

**Figure 6:**
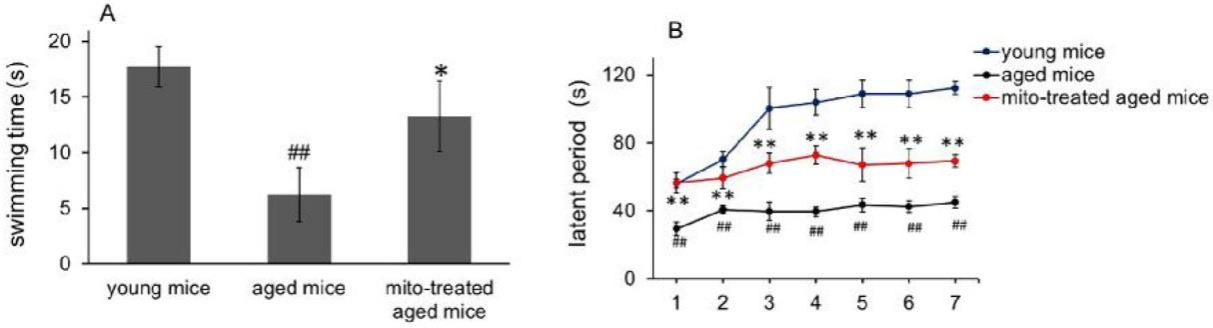
Effects of mitochondria transplantation on swim and rotarod behavior of mice. (A), forced swimming test; (B), rotarod test. N = 10 mice for each group. The group difference was analyzed by ANOVA test. ^##^* p* < 0.01 compared to young control, and *p < 0.05, **p < 0.01 with aged group.

Forced swimming test is one of the most commonly used animal models for evaluation of muscle endurance. The mice treated with the mitochondria showed significantly longer swimming time relative to the control mice, from 7.2 ± 2.4 s to 13.3 ± 3.2 s, suggesting that mitotherapy could increase mouse sport endurance (Fig. 6A).

Then the mice were tested in the rotarod test. The aged mice showed a significant decrease from the second session of the latent period, relative to the control mice (Fig. 6B). However, the mice treated with the mitochondria showed an increase in the latent period from the second session, although the muscle tolerance of the mice was not as good as that of the young mice. These data suggest that the mitochondria could improve sport performance of aged animals.

### Mitotherapy increased phagocytosis of macrophages

**Figure 7:**
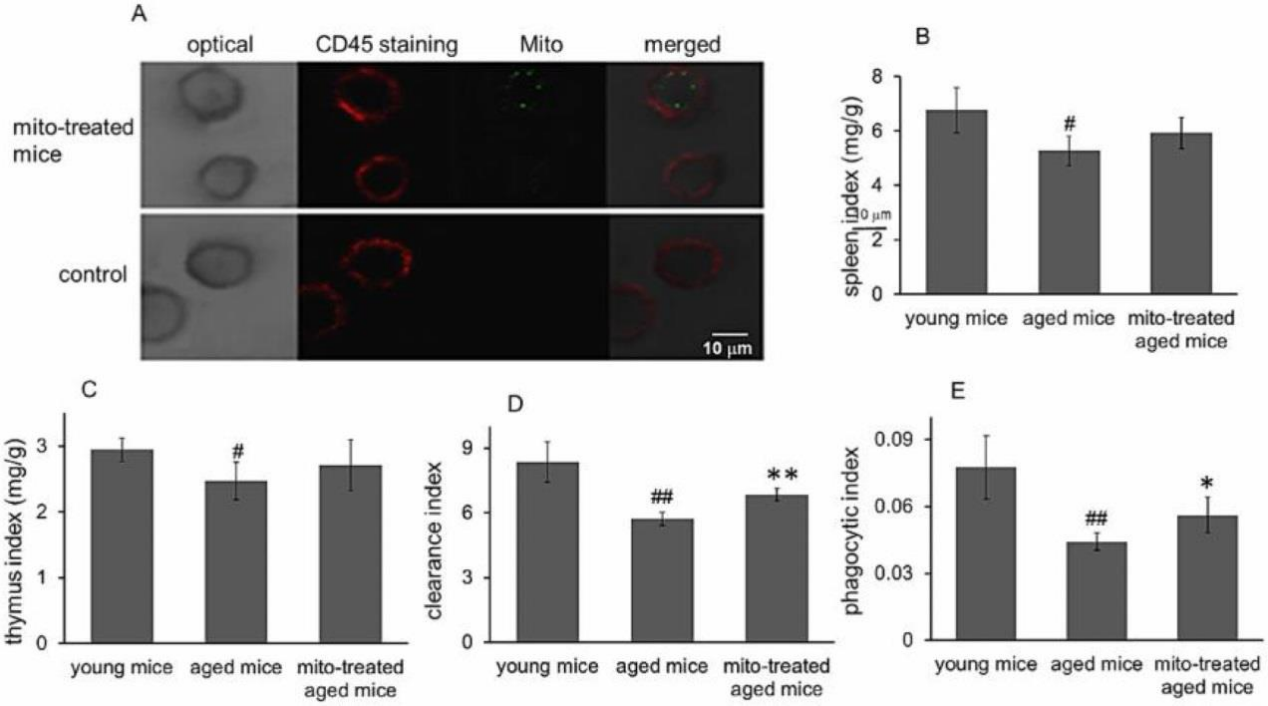
Effects of mitochondria transplantation on phagocytic activity of macrophages. (A), the representative images of CD45 immunofluorescence staining. The green fluorescence-labeled mitochondria could enter the leukocyte. (B), spleen index; (C), thymus index; (D), clearance index; (E), phagocytic index. N = 4 for each group. The difference was analyzed by Student's *t* test. ^#^ *p* < 0.05, ^##^ *p* < 0.01 compared to young control, and *p < 0.05, **p < 0.01 with aged group.

Besides cognitive and motor performance, ATP-dependent immune responses of macrophages reduce in aged animals 15,16, which are particularly at risk for developing chronic diseases. We have identified that exogenous mitochondria are not able to enter red blood cells because the cells lack endocytic function 17. Here CD45 immunofluorescence staining was performed to examine whether the mitochondria could play roles in leukocytes (CD45-positive cells). The results showed that fluorescence-labeled mitochondria could arrive in CD45+ cells, including macrophages (Fig. 7A). Therefore, we further determined the effect of mitotherapy on phagocytosis of macrophages.

Carbon particle clearance test is a common used method to examine phagocytosis function of blood macrophages. Clearance index and phagocytic index significantly decreased in the aged mice, while the mitotherapy partly restored the phagocytic ability of macrophages in aged mice (Fig. 7B and 7C), while no significant changes were observed in the liver and spleen index (Fig. 7D). The results suggested that mitotherapy could stimulate macrophage activation, leading to enhance cellular immunity to exogenous particles.

## Discussion

Mitochondria are cells' powerhouse that is indispensable for energy production and cell survival. One of the most highly investigated theories of aging is the mitochondrial theory of aging 18, which contains a central principle that decreased mitochondrial function is closely associated with aging. Here, we suggest that mitotherapy can supplement bioenergy and reduce the oxidative stress, as well as decrease the ratio of deletion and wild-type mtDNA, leading to improvement of cognitive and motor performance in aged mice. Intriguingly, the mitotherapy can activate the phagocytic activity of macrophages. The reversal effect of the mitochondria on aging provides new evidence to support the mitochondrial theory and opens a novel avenue for anti-aging.

Accumulation of mtDNA mutations would deteriorate respiratory chain function that underlies cell and tissue aging. It is well known that mtDNA encodes 37 genes, including 22 tRNA, 2 rRNA, and 13 protein components of oxidative phosphorylation. Differently from nuclear DNA, the mtDNA lacks damage-repair mechanisms, and the mutations are susceptibility to accumulate. A recent study identified that the onset of aging symptoms would be determined by the ratio of mutant to wild-type mtDNA, with a typical threshold effect 19, and thus the mice with a high mutant ratio of mtDNA exhibit advanced aging phenotypes. Although aging-related mtDNA mutation is regarded as incurable and has to wait for the development of genome editing techniques, the supplement of healthy mitochondria will have great promise to decrease the mutant ratio of mtDNA and then slow down the aging phenotypes.

The mitochondrial theory of aging holds that a decrease of ATP supply and an increase in ROS level cause the damages of cell components, resulting in senescence 20,21. Cellular energy is mainly produced through oxidative phosphorylation taking place within mitochondria. Mitochondria in young animals can produce sufficient energy along with eliminating successive ROS. However, mitochondria in aged animals are characterized by reduced oxidative phosphorylation, increased ROS level, and diminished anti-oxidant defense. Much of the evidence have suggested the ROS produced by mitochondria damage cellular component and induce cell injury, which subsequently causes aging and death 22,23. Therefore, scavenging ROS with antioxidants was recognized as an effective strategy to prevent aging and age-related diseases. However, increasing studies reveal that antioxidants do not reduce animal mortality 24,25, and some of them, such as β-carotene, vitamin E, and higher doses of vitamin A, can increase mortality, since low concentration of ROS caused by antioxidants inhibit ROS-mediated cellular signaling pathway 26. Differently from the anti-oxidants, healthy mitochondria play an important role in maintaining ROS homeostasis through producing and scavenging ROS. Mitochondria coupling with the electron leak in respiratory chain produces ROS, meanwhile mitochondrial enzymatic and non-enzymatic anti-oxidant systems, such as superoxide dismutase and GSH, rapidly eliminate ROS 27,28. In addition, accumulating evidence identify that the exogenous mitochondria could decrease ROS level and increase GSH content after mitochondrial administration, which could be the biochemical mechanisms of recovering cell function and restoring cell viability 7,29.

Here, we used an organ of central nervous system (brain) and a peripheral tissue (skeletal muscle) to study the effect of mitotherapy, since the most obviously and commonly recognized features of aging is energy decline in brain and skeletal muscle function that affects every aspect of human life, such as learning and memory, exercise, posture maintenance. In addition, the brain is a highly energy-consuming organ that requires about 20% of body oxygen with high oxidative phosphorylation to fulfill its function 30. Thus, it is not surprising that mitochondrial dysfunction can cause disturbances in brain energy metabolisms, leading to the decreases in learning and memory ability (one common feature of aging and neurodegeneration diseases). In the previous study, we have suggested that mitotherapy could prevent Parkinson's disease in an experimental mouse model 17. Besides, a current report confirms that exogenous mitochondria following intravenous administration can cross the blood-brain barrier (BBB) to treat depression in mice 31. Although the mechanism of penetrating BBB of mitochondria remains unclear, it might be ascribed to a transcytosis pathway, which is a fundamental biological process used by macromolecules and particles to cross BBB 32. The microvesicles containing mitochondrion may be formed during the transcytosis process. When the microvesicles enter the cells, the fusion of the vesicle membrane with the cytosol membrane would potentially make the intact mitochondria into the cytosol 33. The entry mechanism of mitochondria into cells would be associated with actin-mediated endocytosis because actin polymerization inhibitors can prevent the internalization of mitochondria by cells 34,35. After the mitochondria enter cells, they are transported to lysosomes, then majority of the mitochondria can escape from the lysosomes and play roles in cytosol 36. Moreover, it has been identified that exogenous mitochondria can promote neurogenesis, and activate the expression of brain-derived neurotrophic factor (BDNF) in the reported articles 7,37,38. Here we further identify the organelle therapy improve the cognitive and motor performance in aged animals, suggesting that the mitochondria could be used as a promising candidate to treat aging and age-related diseases.

Moreover, it has been well established that innate immune responses are impaired with aging 37. Recent studies emphasize that mitochondria act as an indispensable player in regulating innate immune and inflammatory responses 38,39. In macrophages of aged mice, mitochondrial dysfunction decreases ATP production, enhances oxidative stress and diminishes antioxidant responses, which will augment tissue damages, and increase morbidity and mortality of older animals in response to pathogens 40. Treatment of the aged macrophages with mitochondrial protector (such as anti-oxidants, NAD^+^) would restore oxidative phosphorylation and homeostatic immune responses. Therefore, in this study, we investigate the effect of mitotherapy on the macrophages, and the results suggest that the mitochondria increase phagocytosis of phagocytes to exogenous particles in aged mice, indicating that mitochondria would enhance the cellular immune responses to pathogens and damaging ligands.

In summary, mitochondria are today in the scientific spotlight and sure hold promises for the future aging research. Here we first find that young mitochondria can partly reverse the age-related behaviors, and the anti-aging mechanism is associated with the control of redox and bioenergy productions. That is, the young mitochondria could augment the functions of dysfunctional mitochondria of aged tissues. Since mitochondrial dysfunction is a common feature in aging and other various diseases, the results provide an insight into the strategy to treat and prevent aging and related diseases through the mitotherapy.

## Materials and Methods

### Animals

Male BABL/c mice with the age of 2 months (young mice) and 18 months (aged mice), were used in the study. The mice were provided by the Animal Breeding Center Affiliated to Chongqing Medical University, China, and were housed under conditions of natural illumination with food and water available ad libitum. The animal experiments were performed following the Chinese Guides for the Care and Use of Laboratory Animals.

### Mitochondrial isolation

Liver mitochondria of young mice were isolated according to the previous report 41. Briefly, young mice were euthanized by cervical dislocation, and then the liver was dissected out immediately. The liver was washed by cold PBS (0.01 M, pH 7.4), then was cut into pieces. The samples were homogenized in cold isolation buffer. Then the homogenate was centrifuged at 800 g for 5 min at 0 ~ 4°C. The supernatant was collected and resuspended in the isolation buffer for another centrifugation at 10 000 g for 10 min. The mitochondria precipitate was washed with the isolation buffer for 2 times. The mitochondria were extraction before use. The numbers of mitochondria were calculated with a counting plate under an optical microscope (Olympus, Tokyo, Japan). Mitochondrial concentration was determined by BCA assay.

### Group assignment

The aged mice were randomly divided into two groups. The mice in one group were slowly injected the saline suspension of the isolated mitochondria via tail veins once in two days (5 mg/kg body weight) for 5 times. Each mouse was injected with 10^7^ mitochondria at a time. The mice in another group were injected same volume of saline (vehicle). Young mice with saline injection were used as the young control. The mice were assigned as n = 4 in each group for every biochemical analysis, and n = 10 in each group for every animal behavior test.

### Quantification of deleted mtDNA in aged mice

The competitive PCR analysis was performed in order to examine the percent of deleted mtDNA to wild-type mtDNA. Mitochondrial DNA (mtDNA) of mouse tissues (brain, skeletal muscle, liver, kidney, lung, heart) was respectively extracted as previously described 42. Three primers, F1 (5' TCTATTCATCGTCTCGGAAG 3'), F2 (5' TACC ATTCCTAACAGGGTTC 3') and R (5' TTTATGGGTGTAATGCGGTG 3'), were synthesized by the Beijing Genomics Institute (Beijing, China). Primer F1/R was used to amplify the deleted mtDNA fragment, while F2/R to wild-type mtDNA sequence 43,44. All of the three primers were added to the reaction system for PCR. Running program of PCR was initiated from 95°C, incubating for 5 min, followed by 40 cycles of 95°C for 10 s, 56°C for 10 s, 72°C for 10 s. Then the products were subjected to 2% agarose electrophoresis in TAE buffer (0.04 M Tris-acetate, 0.001 M EDTA, pH 8.0) at 80 mV for 10 min. The DNA bands were observed under Gel Doc XR (Bio-Rad, USA).

Quantitative PCR (qPCR) was carried out by using ABI ViiA7™ real-time PCR system (Applied Biosystems, Carlsbad, CA, USA) with a SYBR green assay (Perfect Real Time, Takara, Japan). Primers of F1, F2 and R were added into the reaction system, and *β-actin* gene was used as the internal control to normalize the relative levels. The percent of deleted mtDNA was calculated as deleted mtDNA/wild-type mtDNA ×100%.

### Transmission electron microscope observing and biochemical index assay

Mice in the three group were euthanized with 10% pentobarbital sodium after 10 days' injection. Afterward, the mice were transcardially perfused with ice-cold PBS to move out blood, and then mouse brain and skeletal muscle were dissected out for transmission electron microscope (TEM) observing according to the operation manual.

In addition, the mitochondria were isolated from brain and skeletal muscle, and the mitochondrial activities were measured respectively by the resazurin method (Zhang et al., 2004).

Besides, the activities of pyruvate dehydrogenase, α-ketoglutarate dehydrogenase, and NADH dehydrogenase, as well as levels of ATP, reactive oxygen species (ROS), malondialdehyde (MDA) and glutathione (GSH) in homogenates of brain or skeletal muscle, were respectively determined by using individual commercial kits (Nanjing Jiancheng Biotech. Ltd. Co., Nanjing, China), in which Pyruvate dehydrogenase activity was measured by 2,6-dichlorophenol method, α-ketoglutarate dehydrogenase activity was determined through calculation of the product amount of NADH, and NADH dehydrogenase was detected by enzyme-linked immunosorbent assay (ELISA). Moreover, ATP level was measured by spectrophotometer method, and DCFH-DA (Sigma-Aldrich Co.) was used to determine the level of ROS. Thiobarbituric acid (TBA) and 5,5-dithiobis(2-nitrobenzoic acid) (DTNB) were respectively performed to measure MDA and GSH content. Three independent experiments were performed for each assay.

### Forced swimming test

Forced swimming test was carried out in an acrylic plastic pool (50 cm × 50 cm × 40 cm) filled with water maintained at 25 ± 2 °C. The water in the pool was 30 cm deep. Before the test, each mouse's tail was loaded with galvanized wire that was 10% of its body weight, to standardize the workload and reduce the swimming time 45. The swimming time of each group was averaged, and data of the different groups was analyzed.

### Rotarod test

Rotarod test was operated in a rotarod apparatus (Chengdu Taimeng Biotechnology Co., Ltd., Chengdu, China) consisted of a rotating rod, and five individual compartment that was able to test five mice simultaneously 46. Mice were placed on a rod that accelerated smoothly from 5 to 20 rpm throughout 120 s. The time that each animal stayed on the rod was recorded as the latent period. The experiment was repeated three times for each animal at 10 min rest intervals to prevent stress and fatigue.

### Water maze test

The Morris water maze apparatus (Chengdu Technology & Market Co. LTD, China) was used to test spatial learning and memory 47. For navigation test, there were four trials per session and two sessions per day, with one session given in the morning and the other in the afternoon. A total of six sessions in 3 days were given. In each of the four trials, the animals were placed randomly at four different starting positions at the junction between two adjacent quadrants (the east, north, west or south poles of the water maze). The animals were allowed 120 s to find the platform. If an animal could not find the platform in 120 s, it was guided to the platform. The animals were allowed to stay there for 30 s. The time that an individual mouse spent to reach the platform was recorded as the escape latency (s).

Probe test was performed 24 h after the navigation test completed. The platform was removed from the pool, and the mice started from a unique starting location directly opposite the platform. Mice were allowed in the pool for 90 s. All trials were recorded with a digital camera using the computer software of Water Maze. Time spent and distance swam in the target quadrant was recorded.

### Immunofluorescence staining of CD45

The isolated mitochondria were labeled by green fluorescence protein as the previous report 14. After mice were injected the mitochondria for 1 h, the mice were anesthetized, and blood was collected from the heart into Vacutainer® Heparin Tubes. Leukocytes were separated using the commercial kit (Beyotime Biotech. Co., Beijing, China) according to the manufacturer's protocol of leukocyte isolation kit (Beijing Solarbio Science & Technology Co., Ltd., China). The leukocytes were fixed by 70% ethanol and then incubated in 10% normal goat serum that was diluted in PBS at 4ºC overnight. For CD45 recognition, a rabbit monoclonal anti-CD45 antibody (1:1000; Beijing Boaosen Biotch. Co., China) was used, and Texas red-labeled goat anti-rabbit IgG (Shanghai Yeli Biotech. Co., China) were used as secondary antibodies (Ailing et al., 2008). PBS was used to wash the cells before each addition. The cells were air-dried, and placed on coverslips using a fluorescent mounting medium. CD45-positive cells were observed under a confocal microscope (Zeiss, Jena, Germany).

### Evaluation of the phagocytic activity of macrophages

The phagocytic index of the macrophages was determined using carbon particle clearance test. The mice in the three group were intravenously injected the Indian ink (Shanghai Sangon Biotech Co., Ltd., Shanghai, China) with a dose of 0.1 mL/10 g body weight. Blood samples were drawn through retro-orbital plexus into the Heparin Tubes at 0 and 15 minutes after injection. The blood samples (25 μL) was mixed with 3 mL of sodium carbonate, and then optical density was measured at 650 nm using a microplate reader (Bio-Rad, USA). The clearance index (K) was calculated as (log OD_1_ - log OD_2_) / (t_2_ - t_1_), where OD_1_ and OD_2_ are optical densities at 0 min and 15 min. and t_2_ - t_1_ is referred to the time intervals at 0 and 15 minutes. Meanwhile, mouse liver, thymus, and spleen were respectively dissected out and weighed. The ratio of thymus/spleen weight and body weight was respectively calculated as thymus/spleen index. The phagocytic index was represented as [body weight/(liver + spleen)] ×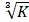.

### Statistical analysis

Data were presented as mean ± standard error of the mean (S.E.M) of three independent experiments. Statistical product and service solutions (SPSS) 13.0 software was employed in the data analysis. Significant differences were determined by independent sample t-test or one-way analysis of variance (ANOVA), following by a post hoc test (Tukey's method). Differences were considered significant when *p* < 0.05.
